# Expert recommendations to personalization of medical approaches in treatment of multiple sclerosis: an overview of family planning and pregnancy

**DOI:** 10.1186/1878-5085-3-9

**Published:** 2012-06-22

**Authors:** Nadja Borisow, Andrea Döring, Caspar F Pfueller, Friedemann Paul, Jan Dörr, Kerstin Hellwig

**Affiliations:** 1 NeuroCure Clinical Research Center and Clinical and Experimental Research Center for Multiple Sclerosis, Charité - Universitätsmedizin Berlin, Charitéplatz 1, Berlin 10117, Germany; 2 Department of Neurology, St. Josef Hospital, Ruhr University Bochum, Bochum 44801, Germany

## Abstract

Multiple sclerosis is the most common chronic autoimmune disease of the central nervous system which preferentially affects females at childbearing age. For this reason, patients and treating physicians were frequently confronted with questions concerning family planning, pregnancy and birth. Preventive and personalized treatment approaches are considered, because topics as heredity, risk of congenital malformations, influence of pregnancy on MS and aspects of drug therapy during the period of conception, pregnancy, puerperium and lactation have to be discussed. Here, we provide an overview about the current state of knowledge regarding these issues.

## Review

### Introduction

Multiple sclerosis (MS) is a chronic autoimmune disease of the central nervous system which, at the beginning, typically arises with relapsing episodes of neurological deficiencies. Subsequently, symptoms may persist, and in approximately 65% of the patients, a conversion into a secondary progressive (SP) course with a continuous deterioration of disability occurs. Patients (15-20% of the patients suffer from a primary progressive (PP) MS with a steady worsening of symptoms from onset [1]).

Until now, pathomechanisms of MS are not sufficiently understood. Inflammatory demyelinating as well as neuroaxonal degenerative processes are involved in pathogenesis and result in cerebral and spinal tissue damage [1].

Treatment of acute exacerbation includes usually the application of intravenous methylprednisolone for 3 to 5 days [2,3]. To reduce frequency and severity of relapses as well as the progression of disease in general, an immunomodulatory therapy with interferon beta (IFN-β) or glatiramer acetate (GA) is necessary [4,5]. In patients with high disease activity or insufficient effect of IFN-β or GA, the monoclonal antibody natalizumab or the sphingosine receptor modulator, fingolimod, is available [6,7]. Another agent, which is approved for second-line-therapy, is mitoxantrone [8]. Of particular importance to reduce relapses and disease progression is an early initiation as well as a long-lasting and continuous administration of these therapies [9,10].

In Europe, MS prevalence rates range between 10 and 187 per 100,000 with a higher rate in northern countries [11]. In Germany, approximately 120,000 people suffer from MS [12]. Patients typically experience first symptoms between the 3rd and 5th decade of life, and thus, MS mainly affects patients in childbearing age. It shows a female predominance of 2 to 3:1 [11-13]. Consequently, a high need for counseling exists on topics as family planning, pregnancy and child birth. Seeing that even healthy couples have many concerns related to pregnancy, such concerns particularly apply to patients with chronic and progressive diseases such as MS.

As especially the field of family planning, pregnancy and child birth benefits from a shift from delayed interventional to preventive and personalized medicine, in this article, an update about the current state of knowledge regarding the process of decision making, heredity and fertility, as well as course and outcome of pregnancy, in MS is given. Furthermore, we want to discuss the influence of pregnancy and childbirth on the disease, aspects of breastfeeding and concomitant medication, and finally encourage MS patients not generally to abstain from child birth due to their disease.

### Influence of MS on family planning and personalized approach in decision making

In patients with MS, a lot of different aspects and concerns influence the decision whether to bear a child or not [14]. In the past, especially during the first half of 20th century, female patients were often discouraged from becoming mother as pregnancy was considered to be a risk in MS [15]. Today, first and foremost, there is the fear about the future state of health and the associated ability of taking care for the baby. The unpredictable course of the disease and the known increase of relapses postpartum [16] lead to further uncertainty regarding getting pregnant. Disabilities, e.g., pareses or impaired coordination may result in a higher risk of injury for the infant. Further anxiety exists regarding genetic predisposition and passing the disease on to their offspring. Moreover, the necessity to pause virtually all established disease-modifying therapies during the period of conception, pregnancy and breastfeeding raises the possibility of disease progression. Frequently, patients feel pressured by family members or health professionals either to abstain from pregnancy due to various problems MS is related with or to bear children as soon as possible in an early stage of disease. The fear of asking others for help and finally financial restrictions are other aspects patients with MS are confronted with while planning a family. In a widely larger extent than in healthy women in patients with MS, other persons besides the partner are involved in the process of decision making regarding getting pregnant [17]. The patients try to get into conversation especially with other family members, foremost their mothers, as they are aware they could rely on them in case of a relapse. Depending on the relationship and the sense of togetherness within the family, these talks may be encouraging as well as dissuasive. Also, treating physicians were frequently consulted to achieve an additional advice from the medical point of view.

Several studies were published regarding the correlation between MS and family planning, especially child birth, to some extent with contradictory results. A French study revealed a relationship between age at onset of the MS and the number of children who are finally born [18]. A clear association between an early disease onset (<25 years) and a lower number of children could be shown, even though patients with an onset >25 years get less children than the general French population. Interestingly, there is no difference between men and women [18]. A Swedish study shows that, even at onset of the disease, the proportion of childless women is higher in female MS patients compared to healthy women of same age [19]. The reasons are not clear; maybe a reduced fertility even before onset of the disease has to be discussed.

### MS inheritance risk as factor influencing individual decision making in family planning

From the perspective of preventive medicine, knowledge about inheritance risk is essential for making informed decision about planning or continuing a pregnancy. Broad consensus exists that environmental as well as genetic factors are involved in the etiology of MS. The lifetime risk for MS in the general population of northern Europe is 0.2% to 0.5% with higher rates in northern countries. As epidemiologic studies show, risk of MS increases with raising degree of kinship to a MS patient. Highest risks are described in monozygotic twins, followed by siblings, parents and children of MS patients [20-25]. Second and third degree relatives have a lower risk, although it is still higher than in general population. Among stepsiblings and adopted relatives, no elevated MS risk was found [26,27]. Table 1 gives an overview about studies examining risk of MS in children of male and female MS patients. If not mentioned by the authors, relative risks were calculated by using age-adjusted risks and lifetime risk in general population.

**Table 1:** Overview of studies on MS risk in children of MS patients

**Author**	**Year**	**Country**	**Number of patients**	**Estimated lifetime risk in general population (%)**	**Male MS patients**				**Female MS patients**			
					**Age-adjusted risk for children**		**Relative risk for children**		**Age-adjusted risk for children**		**Relative risk for children**	
Nielsen et al. [20]	2005	Denmark	8205	Men, 0.3; women, 0.5	-		Daughter	7.5	-		Daughter	4.5
							Son	7.5			Son	9.5
							Total	7.5			Total	6.3
Prokopenko et al. [23]	2003	Italy (Sardinia)	313	0.157	-		-		Total	3.19	Total	20.3
Carton et al. [22]	1997	Belgium	674	0.175	Total	1.51	Total	8.6	Total	1.87	Total	10.7
Robertson et al. [21]	1996	UK	674	0.3 (men, 0.13; women, 0.66)	Daughter	7.12	Daughter	10.8	Daughter	1.74	Daughter	2.6
					Son	2.54	Son	19.5	Son	0.0	Son	0.0
					Total	4.91	Total	16.4	Total	0.81	Total	2.7
Sadovnick and Baird [24]	1988	Canada	815	0.1	Daughter	5.13	Daughter	51.3	Daughter	4.96	Daughter	49.6
					Son	0.0	Son	0.0	Son	0.0	Son	0.0
					Total	2.47	Total	24.7	Total	2.58	Total	25.8

To sum up, children of MS patients are at 6- to 12-fold risk of developing MS with age-adjusted risks ranging between 1.5% and 7%. One study even describes a 50-fold risk in daughters of MS patients [24], but all in all, the likelihood of not getting the disease in children of MS patients is approximately 97%.

### Oral contraception - a measure of prevention in MS?

As the intake of oral contraception might play a preventive role in MS, a couple of studies examined the influence of oral contraception on risk, onset and course of MS. In summary, there is no positive proof of an effect of oral contraceptives on the lifetime risk for development of MS [28-30]. In patients with PP MS, the use of oral contraception showed both a higher risk to reach an Expanded Disability Status Scale (EDSS) of 6 and a shorter duration between onset of MS and EDSS 6 [31]. In women with relapsing onset of the disease, no influence of oral contraception on disease progression was found. In contrast to that, another study suggests a protective effect of contraceptives as it shows a correlation between use of oral contraception and a higher age at onset, even though in this study, no differentiation between relapsing and progressive MS was conducted [32]. Another survey as well postulated a possible delay of MS onset in patients using oral contraceptives [33]. Finally, to further evaluate the impact of oral contraception on MS, it seems to be important to differentiate between inflammatory and degenerative processes in the disease course.

### Fertility in MS patients

To explain the higher proportion of childlessness among MS patients, various factors have to be discussed. As mentioned above [19], among others, a reduction of fertility even in the preclinical phase of disease has to be considered. Fertility may be influenced by numerous parameters including sexual dysfunctions and hormonal alternations.

One study in 73 German female MS patients revealed an average period of 4 months to get pregnant and concluded no reduction in fertility in women with MS [34]. However, only females who were already mothers were included in this study which implies that women with limited fertility, leading to childlessness, were not taken into account at all. Thus, this study potentially underestimates the prevalence of reduced fertility in female MS patients. In fact, another survey found a significantly higher need for artificial insemination in female patients with MS compared to the general population obtained from Finnish Medical Birth Register [35].

Even in MS patients with mild neurologic deficits, up to 73% complain about sexual dysfunctions [36,37]. In female patients, reduced libido, decreased vaginal sensation and lubrication as well as difficulties in reaching an orgasm were described [38]. Male patients complain about erective dysfunction, impaired ejaculation, and just as female patients, about reduction of libido [39]. Despite same marital status, a significantly lower frequency of sexual intercourse in patients with MS compared to both healthy controls and patients with rheumatic diseases, especially with rheumatoid arthritis, can be detected [37]. In addition to sexual dysfunctions caused by MS, other disease-related physical impairments may interfere with sexual activity. Above all, fatigue is a frequently mentioned symptom; furthermore, muscle weakness, constraints of coordination, pain, spasticity and impaired sensation may have negative effects on sexuality. In addition, medication used to treat attendant symptoms as depression, anxiety, or urinary urgency may account for sexual dysfunctions. That is why a personalized approach to concomitant medication is required, particularly in MS patients at childbearing age.

Several hormonal alterations which may interfere with fertility have been described in patients with MS. Significantly higher levels of FSH, LH, prolactin and testosterone were detectable in female patients [40]. In men, lower levels of testosterone, LH and FSH, as well as pathologic GnRH and hCG tests, were observed. Above all, pathologic spermiograms with a reduced number and motility of semen were described [41].

Besides these aspects, psychological factors may not be underestimated. Shame because of physical disabilities, bladder or bowel dysfunctions, inserted catheters, or the fear of disapproval by the partner may lead to avoidance of sexual contacts. Whether and to what degree these factors in detail lead to a higher rate of undesired childlessness in MS-patients is not clear. To our clinical experience, fertility is not reduced in a significant way in MS patients; besides this, most women become pregnant in the earlier phase of the disease without profound disability.

### Influence of motherhood on MS

Studies analyzing the influence of pregnancy and child birth on incidence, onset, course, or severity of MS under the aspect of a possibly preventive effect have to be interpreted with caution as cause and effect are difficult to differentiate. A Danish study investigated the relationship between the number of born children and the likelihood of developing MS [42]. The risk of MS was inversely associated with the number of born children and with the age at birth of the first child. Furthermore, the MS risk was reported to increase with time since birth of the most recent child. Moreover, the number of children born before diagnosis of MS is associated with a higher age at onset of the disease [32]. In addition, the pregnancy with or birth of at least two children leads to a lower likelihood to reach an EDSS score of 6, respectively to the extension of the period until EDSS 6 is achieved, at least in patients with relapsing-remitting (RR) MS [31]. If the first child is born after onset of MS, the risk of conversion from RR MS to SP MS is lower compared to women whose children were born before onset of the disease [19]. Furthermore, pregnancy after onset of MS is associated with a longer duration until wheelchair dependence develops compared to patients who were never pregnant or born children only before onset of the disease [43]. To sum up, motherhood does not seem to have negative effects on risk or course of MS, although the interpretations of all these studies need to factor a possible bias as women with severe course of the disease may tend to prevent pregnancies, and rather patients with benign forms or late onset of the disease decide to become pregnant.

### Influence of pregnancy on MS course

The level of knowledge about the effect of pregnancy on MS and vice versa is low in female patients [44], although numerous studies investigated the influence of pregnancy on MS. A prospective study examining the course of pregnancy in about 250 patients, the PRIMS study, revealed a reduction of the relapse rate during pregnancy, especially during the third trimester [16]. Over a period of 3 months after delivery, relapse rate increased to a level higher than before pregnancy followed by a decrease to pre-pregnancy rate during the next 6 to 9 months. These results confirmed the findings of previous partly retrospective, partly quite small studies [45-47] and were verified by following surveys [48,49]. Relapses in the year before and during pregnancy are supposed to be a risk factor as they lead to a 1.7- to 1.8-fold increase of postpartum relapses [50], although these results could not be confirmed by another survey [51].

Immunologic changes during pregnancy aiming at protecting the fetus from maternal immune defense are supposed to lead to the observed reduction of disease activity during proceeding pregnancy. An increase of CD4^+^/CD8^+^ ratio and a decrease of CD16^+^ natural killer (NK) cells during pregnancy, followed by a decline in CD4^+^/CD8^+^ ratio and a rise in CD16^+^ NK cells after delivery may represent a correlate of maternal immune suppression which may explain some of the detected changes in disease activity during and directly following pregnancy [52].

### Assisted reproduction technique in MS patients

As approximately 10% to 15% of all couples in general population remain undesirably childless, artificial fertilization may also be necessary in patients with MS. Until now, three studies investigated the effect of assisted reproduction technique (ART) on the course of the disease. Laplaud et al. found an increased relapse rate in patients treated with LHRH agonists, but not in females after treatment with LHRH antagonists [53]. Two further surveys confirmed the observed elevation in relapse rate during ART, although in these studies, the increased relapse rate was not dependent on the type of hormonal treatment [54,55]. Generally, we do not recommend to refrain from ART but inform about the increased risk and counsel to stay on MS treatment while undergoing ART.

### Multidisciplinary approaches on course and outcome of pregnancy in MS patients

Additional causes of concern in expectant mothers with MS are possible complications during pregnancy or delivery. A study comparing 432 births in 321 MS patients with healthy controls showed no differences in birth weight, mean gestational age, labor duration or 5-mine APGAR score [56]. Some surveys revealed a significant lower birth weight in newborns of MS patients, although the difference was low, ranging between 108 and 123 g [57,58]. A meta-analysis evaluating the results of 22 studies published between 1983 and 2009 found no elevated risk of low birth weight by analyzing the proportion of newborns with a birth weight <2500 g. Furthermore, no increased rate of abortions, prematurity, malformations or neonatal deaths in children of female MS patients was detected [59]. One study in nearly 200 pregnant women with MS provided evidence for a significantly higher rate of maternal anemia which is, due to a chronic intrauterine hypoxia, supposed to be the reason for a higher rate of meconium aspiration in infants of MS patients [60]. Despite these complications, no differences in duration and way of delivery, APGAR score directly after birth or necessity of assisted ventilation were detectable. The frequency of other pregnancy-related complications, such as gestational diabetes mellitus or preeclampsia, was comparable to healthy controls.

In some surveys, operative deliveries with use of forceps or vacuum extraction were more frequent in MS patients [35,57,60], which is attributed to a higher risk of exhaustion and slow progression of delivery in these patients. The rate of deliveries by cesarean section among MS patients shows a wide range between 10% and 40% [59]. In general, proportion of cesarean section in healthy women differs a lot between various countries, influenced by social, religious and cultural factors. Concern regarding present or potential MS-related symptoms during delivery, such as spasticity, neuromuscular perineal weakness or exhaustion, is a factor that may influence the decision of patients and physicians in favor of cesarean section. Delivery mode and use of epidural analgesia do not influence postpartum relapse rate [16]. Taken together, the fact of suffering from MS is no reason to choose cesarean section instead of vaginal delivery, but obstetrical rationale should be the basis for decision making. Multidisciplinary approaches are required to guarantee an optimized treatment.

It has to be taken into account that some cohorts, investigated for pregnancy outcomes, included, first and foremost, women with a low EDSS score [35,56]. Data on rate of pregnancy and delivery complications in women with higher disability are rare, most likely because they are difficult to recruit, as women with distinct deficits rather avoid pregnancies. Furthermore, a higher rate of obesity in women with MS, e.g., due to less activity caused by physical disability, is discussed to possibly elevate the risk of complications during pregnancy and delivery.

### Breastfeeding - a countermeasure against postpartum relapses?

Several studies examined the influence of breastfeeding on the course of the disease, especially on the occurrence and frequency of postpartum relapses, with finally inconsistent results. Some studies postulated a protective effect of exclusive breastfeeding [61-63] with a significantly higher risk for postpartum relapses in women that abstain from nursing. One study described a correlation between a decrease in interferon gamma producing CD4^+^ cells and postpartum relapses, which may be prevented by exclusive breastfeeding due to lactational amenorrhea [64]. On the other hand, the PRIMS study and a number of further investigations found no relationship between breastfeeding and postpartum relapses [16,65,66]. The major difficulty in all these studies is to correctly interpret the causality, as many women stop breastfeeding due to a relapse and treatment with corticosteroids, which may be suggestive of a higher rate of relapses in women that do not nurse at all or only for a short period of time. Except the fact that there is no proof of negative effects of nursing on the course of MS, at the moment, a clear recommendation regarding breastfeeding as a measure for prevention of postpartum relapses cannot be made. The appreciation between early resumption or the beginning of immunomodulatory therapy after delivery on one hand and the beneficial effects of breast milk on the other hand raises a lot of uncertainties. As long as no advantage of breastfeeding on MS course is proven, treating physicians together with the patient have to weigh up the benefit and the risk for relapses by taking into consideration the frequency and severity of disease course before pregnancy to realize an individually tailored and optimized medical care.

### Personalized medication during pregnancy - a challenge in MS treatment

The topic which certainly requires most counseling in patients with MS who wish to conceive is the field of concomitant medication. This contains disease-modifying therapy, immunosuppressive agents and steroids, as well as medication required to treat MS-related symptoms.

As no reliable data concerning safety during pregnancy exist, it is generally recommended to discontinue disease-modifying therapy as IFN-β or GA either at the beginning of unprotected sexual intercourse or at the latest after pregnancy becomes known. Although data regarding drug exposure in human gravidity mainly result from unplanned pregnancies and are usually restricted to the first weeks of pregnancy, they are of particular importance as they help to make personalized treatment decisions in pregnant MS patients.

One study showed a significantly increased rate of spontaneous abortion and a reduction of birth weight after exposure to IFN-β [67]. Reduced birth weight could be confirmed in some surveys [68,69], whereas others found no reduction in birth weight or increased rate of spontaneous abortion, still birth and congenital abnormalities [70-76]. A small survey, including ten patients who continued therapy with IFN-β or GA during whole pregnancy and postpartum, revealed no increased rate of spontaneous abortion but lower birth weight and earlier delivery after GA exposure [69]. Table 2 provides an overview about the results of different studies on birth outcome after exposure to IFN-β and/or GA. To sum up, IFN-β and GA seem to be considerably safe during pregnancy, so an induced abortion after exposure is not mandatory. Regarding the safety of disease-modifying therapy in male MS patients, one only partly prospective study exists which did not show reduced birth weight or length in comparison to babies of healthy women [77].

**Table 2:** Overview of studies on birth outcome after exposure to IFN-β or GA

**Author**	**Number of pregnancies with exposure to immunomodulatory therapy**		**Results**
	IFN-β	GA	
Boskovic et al., 2005 [67]	23		Increased rate of spontaneous abortion (39%); decreased birth weight, two major birth defects (Down syndrome, X-chromosome abnormality)
Sandberg-Wollheim et al., 2005 [73]	41		Normal spontaneous abortion rate, one birth defect (hydrocephalus)
Patti et al., 2008 [72]	14		Normal spontaneous abortion rate and birth weight; shorter gestational period (37.8 weeks), no malformations
Hellwig et al., 2009 [71]	17		Normal miscarriage rate (2/17) and birth weight
Weber-Schoendorfer and Schaefer, 2009 [75]	69	31	Normal spontaneous abortion rate and birth weight, no preterm delivery; two major birth defects (club feet, AV canal) under GA
Amato et al., 2010 [68]	88		Normal spontaneous abortion rate; lower birth weight and length; no major birth defects
Salminen et al., 2010 [76]		13	In 9/13 exposure during whole pregnancy, normal spontaneous abortion rate and birth weight, no birth defects
Fragoso et al., 2010 [74]		11	Exposure >7 months during pregnancy; normal spontaneous abortion rate, birth weight and length
Sandberg-Wollheim et al., 2011 [70]	425		Normal spontaneous abortion rate, three major birth defects (VACTERL syndrome, tetralogy of Fallot, solitary kidney)
Hellwig et al., 2011 [69]	7	3	Exposure during whole pregnancy; lower birth weight and earlier delivery (GA); one malformation in IFN (valvular stenosis of pulmonary artery), one in GA (penile hypospadia)

Regarding the application of natalizumab during pregnancy, approximately 40 patients are reported in the literature, one case with accidental exposure until the third trimester [78-80]. No elevated risks for spontaneous abortion, reduction of birth weight or lengths especially congenital malformation were found; however; due to the sparse data it is not possible to rule out rare complications.

As animal studies in fingolimod showed increased rates of miscarriage and vascular malformations, its use during pregnancy is not recommended, although experience in human pregnancy is missing.

Glucocorticoids are not considered to be safe during the first 3 months of pregnancy, as an increased risk of oral clefts cannot be excluded [81]. Furthermore, higher rates of miscarriage and preterm births, as well as lower birth weight, have been described [82]. In cases of severe relapses glucocorticosteroids can be taken into account individually. Beyond the first trimester, the use of glucocorticoids does not seem to be harmful to the baby [83].

In MS, chemotherapeutic agents are used as second- or third-line therapy in highly active relapsing-remitting MS or in progressive courses, but due to a high rate of side effects, their use is limited. They are, in general, not recommended during pregnancy, partly because of known teratogenicity, partly due to missing data regarding possible effects on and risks for embryo- or fetogenesis in human. Methotrexate, as well as other chemotherapeutics, is contraindicated during pregnancy, as it leads to spontaneous abortions and congenital malformations [84]. According to manufacturers' information, cyclophosphamide is mutagenic; in the case of an exposure during first trimester of pregnancy, counseling regarding induced abortion should be provided. The FDA classifies cyclophosphamide within category D, which means that fetal risks are known, but in individual cases, the potential benefit may outbalance the risk. Two surveys report the neonatal outcome after mitoxantrone exposure during pregnancy in MS patients; one of them describes growth restriction without evidence of congenital malformation [85], and in another case, a child with Pierre Robin sequence was born [86]. Mitoxantrone is also classified within category D by FDA. In azathioprine, until now, no teratogenic effect was reported in humans; nevertheless, it should only be used after strict risk-benefit analysis because, in some newborns, leuko- and/or thrombopenia were described.

Intravenous immunoglobulin (IVIG) is supposed to be safe during pregnancy as no negative effects on gravidity or fetal development have been reported. A retrospective study found a significant reduction of relapse rate by IVIG administration during pregnancy and postpartum without any serious adverse effects [87]. However, IVIGs are not approved for MS treatment in general, and not much information about the efficacy for acute relapses is available.

Table 3 summarizes FDA pregnancy classification of different immunomodulatory and immunosuppressive medication used in MS therapy.

**Table 3:** FDA pregnancy categories

**FDA pregnancy category**	**Description**	**Drug**
**B**	Animal studies have revealed no evidence of harm to the fetus; however, there are no adequate and well-controlled studies in pregnant women.	Glatiramer acetate
	Or	
	Animal studies have shown an adverse effect, but adequate and well-controlled studies in pregnant women have failed to demonstrate a risk to the fetus in any trimester.	
**C**	Animal studies have shown an adverse effect and there are no adequate and well-controlled studies in pregnant women.	IFN-β, natalizumab, fingolimod, dexamethasone, prednisolone, IVIG and cyclosporin A
	Or	
	No animal studies have been conducted, and there are no adequate and well-controlled studies in pregnant women.	
**D**	Adequate well-controlled or observational studies in pregnant women have demonstrated a risk to the fetus. However, the benefits of therapy may outweigh the potential risk. For example, the drug may be acceptable if needed in a life-threatening situation or serious disease for which safer drugs cannot be used or are ineffective.	Cyclophosphamide, azathioprine and mitoxantrone
**X**	Adequate well-controlled or observational studies in animals or pregnant women have demonstrated positive evidence of fetal abnormalities or risks. The use of the product is contraindicated in women who are or may become pregnant.	Methotrexate

Medication to treat MS-associated symptoms, e.g., oxybutynin in neurogenic bladder dysfunction, spasmolytics as baclofen and tizanidine, or antidepressants such as amitriptyline, citalopram or mirtazapine should only be used after careful consideration between risks for the unborn and gain for the mother. A detailed discussion on supportive treatment options in the context of pregnancy is, however, beyond the scope of this article.

### Prevention of postpartum relapses

Especially the first months after delivery pose a challenge for the newly minted parents and are particularly important for the relationship between mother and child. Unfortunately, just in the first 3 months after child birth, the relapse rate in MS patients markedly increases. Thus, finding an effective prevention for postpartum relapses is a matter of particular importance. Based on the hypothesis of a progestin-mediated immunological shift from Th1 to Th2 response, which is considered an important cause of reduced relapse rate during pregnancy, the POPART'MUS study examines the potential of progestin and estradiol in preventing postpartum relapses [88]. It is a prospective, placebo-controlled and double-blinded study that aims to recruit 300 patients and is still ongoing.

Intravenous corticosteroids are supposed to reduce postpartum relapse rate [89]; however; until now; prospective randomized double-blinded studies are missing. A postpartum therapy with intravenous immunoglobulin as well reduced the number of relapses [62,90]. Moreover, in contrast to IFN-β or GA, breastfeeding is possible while receiving IVIG. If treatment with IVIG already started during pregnancy, an even additional reduction of relapse rate may be achieved [87]. Finally, further large scale studies, preferably placebo controlled, are required to confirm the positive effects of glucocorticoids and IVIG in the prevention of postpartum relapses.

### Being parent with MS

Many expectant mothers with MS ask themselves if they will be able to deal with everyday life after delivery and if despite their disease they will be able to be a good mother. An important role in the daily routine of newly minted mothers with MS plays the economizing of their physical resources. As exhaustion affects even healthy mothers during the first months after delivery, it impacts mothers with MS in a particular degree. Interviews with mothers with MS show that they pass on to a forward-looking planning of daily household activities which markedly alleviates to conserve their energies [17]. Female patients with children seem to have a higher quality of life compared to childless MS patients, at least concerning the domain of social function [91]. On the other hand, in mothers with MS, the concern for children correlates with depressive symptoms, but social support may at least partly alleviate them [92]. Finally, being a good mother and taking care for a baby is also possible with a disease like MS, although the social support is even more important than for healthy mothers.

## Conclusions

A lot of questions arise if patients with MS get pregnant or plan to conceive. It is important not to discourage these women but to comprehensively inform about possible risks and specific features of pregnancy in MS. These include a slightly elevated risk for heredity, the possibility of disease-related sexual dysfunctions and an increase of relapse rate during puerperium. On the other hand, specific complications during pregnancy or congenital abnormality are not to be feared for. Patients have to be aware of limited knowledge about the effect of various MS medication on pregnancy as well as that, until now, an unambiguous recommendation of breastfeeding is not possible. Thus, a weighting between risk and benefit is frequently necessary, which should always be done after detailed counseling and together with an experienced physician. Prevention of postpartum relapses poses a challenge for the future to encourage even more patients to fulfill their desire to start a family.

## Competing interests

The authors declare that they have no competing interests.

## Authors’ contributions

NB performed literature search, identified relevant studies and wrote the final review. JD and KH acted as review authors and provided content expertise. FP, CP and AD provided further content expertise and read and commented on drafts. All authors read and approved the final manuscript.
